# Supplementary material for: Enhanced Reactive Brilliant Blue Removal Using Chitosan–Biochar Hydrogel Beads
Source: Molecules. 2023 Aug 19;28(16):6137. doi: 10.3390/molecules28166137 (PMC10458918; doi:10.3390/molecules28166137)
Supplement: Supplementary file 1 [file molecules-28-06137-s001.zip › molecules-2547033-supplementary.pdf]

Supplementary Materials

Enhanced Reactive Brilliant Blue Removal Using Chitosan–Biochar Hydrogel Beads

Yangyang Zhao [1,*], Yang Song [1], Rui Li [2], Fengfan Lu [1], Yibin Yang [1], Qiongjian Huang [1], Dongli Deng [1], Mingzhu Wu [1] and Ying Li [1,*]

[1] Chemical Pollution Control Chongqing Applied Technology Extension Center of Higher Vocational Colleges, Chongqing Industry Polytechnic College, Chongqing 401120, China; songyang@cqipc.edu.cn (Y.S.); luff@cqipc.edu.cn (F.L.); yangyb@cqipc.edu.cn (Y.Y.); huangqj@cqipc.edu.cn (Q.H.); dengdl@cqipc.edu.cn (D.D.); wumz@cqipc.edu.cn (M.W.)

[2] School of Biological Science, Jining Medical University, No. 669 Xueyuan Road, Donggang District, Rizhao 276826, China; ruili061289@163.com

* Correspondence: zhaoyy@cqipc.edu.cn (Y.Z.); liying@cqipc.edu.cn (Y.L.)

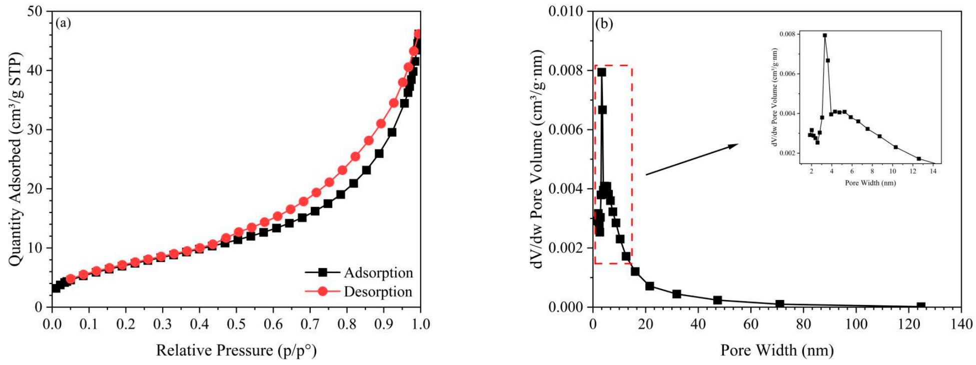

Figure S1. (a) Isotherm linear plot of CBHBs; (b) BJH desorption dV/dw pore volume plot of CBHBs.
